# Pharmacokinetics of Single-Dose Oral Pregabalin Administration in Normal Cats

**DOI:** 10.3389/fvets.2018.00136

**Published:** 2018-07-20

**Authors:** Michaela A. Esteban, Curtis W. Dewey, Wayne S. Schwark, Mark Rishniw, Dawn M. Boothe

**Affiliations:** ^1^Department of Clinical Sciences, College of Veterinary Medicine, Cornell University, Ithaca, NY, United States; ^2^Department of Molecular Medicine, College of Veterinary Medicine, Cornell University, Ithaca, NY, United States; ^3^Department of Clinical Sciences, College of Veterinary Medicine, Auburn University, Auburn, AL, United States

**Keywords:** pregabalin, cats, seizures, pharmacokinetics, epilepsy

## Abstract

**Objective:** To describe the pharmacokinetic parameters of oral pregabalin in normal cats after single oral dosing.

**Animals:** Six healthy adult research cats.

**Procedures:** Following sedation and indwelling catheter placement, one oral (4 mg/kg) dose of pregabalin was administered. Blood samples were collected at 0, 15 and 30 min and 1, 1.5, 2, 3, 4, 6, 8, 12, 24, and 36 h after administration. Plasma pregabalin concentrations were measured by high-performance liquid chromatography and subjected to pharmacokinetic analysis using commercial software.

**Results:** Four of six cats developed moderate sedation after pregabalin administration. The peak pregabalin concentration was 8.3 ± 1.6 μg/ml which occurred at 2.9 ± 1.2 h. Elimination half-life was 10.4 ± 2.6 h and area under the curve was 133.9 ± 71.5 μg-h/ml. Time above the minimum therapeutic concentration for seizure control in dogs and people (2.8 μg/ml) was 17.6 ± 6.2 h. Using these data, predicted minimum, maximum and average steady state concentrations were calculated for 12 and 24 h dosing intervals.

**Conclusions and Clinical Relevance:** Pregabalin (4 mg/kg) administered orally to cats results in plasma concentrations within the range considered to be efficacious for seizure control in dogs and humans between 1.5 and at least 12 h. Because of moderate sedative side effects in the majority of cats at this dose and high calculated maximum steady state concentrations, a lower dose, given more frequently (1–2 mg/kg q 12 h), should be evaluated in prospective clinical studies.

## Introduction

Seizure disorders in cats currently have few treatment options, with most clinicians prescribing phenobarbital to manage seizures. However some cats are refractory to phenobarbital and require additional anticonvulsants for adequate seizure control; others exhibit unacceptable side effects (1, 2). With the exception of levetiracetam, few anticonvulsant medications are considered safe and effective for cats with seizure disorders (3).

Pregabalin, (S)-3 aminomethyl-5-methylhaxanoic acid, is a neuro-active drug that, like gabapentin, inhibits the α2-δ auxiliary protein subunit of voltage-gated calcium channels (4). Binding to the subunit reduces calcium influx at nerve terminals, preventing release of neurotransmitters such as glutamate, norepinephrine and substance P. Although pregabalin is a γ-aminobutyric acid (GABA) analog, it does not exhibit any GABA-ergic activity. Pregabalin shows higher affinity to the α2-δ protein subunit than gabapentin (5), suggesting that pregabalin is more potent than gabapentin.

Pregabalin has been shown to be effective and well-tolerated as an add-on anticonvulsant medication in people with partial (focal) seizures (6, 7), and for management of neuropathic pain, fibromyalgia, peripheral neuropathy (8), post herpetic neuralgia (9, 10), and generalized anxiety disorders (11). In humans it displays near complete oral absorption and is eliminated via the kidneys without significant metabolism (greater than 90% excreted unchanged) (4). Humans with renal insufficiency demonstrate decreased clearance (12), but the drug appears free from drug-drug interactions, making it an attractive add-on medication for seizure control.

A recent study described the pharmacokinetic parameters of gabapentin in cats after both oral and intravenous administration (13). A three-compartment model was used to fit intravenously administered gabapentin, and a one-compartment model was used to fit oral administration data. Half-life for orally administered gabapentin was 177.2 min and it was concluded that four times daily oral dosing was necessary for optimum clinical efficacy. Similarly, the pharmacokinetics of pregabalin, and its effectiveness as an add-on anticonvulsant medication in dogs, have been reported (14, 15). Pregabalin administered at 4 mg/kg was well tolerated and achieved blood concentrations considered therapeutic for human epilepsy between hours 1–8 after administration (14). In dogs, pregabalin had half-life of 6.8 h, and time over the presumed minimum effective concentration of 10.8 h. A prospective clinical trial evaluating the efficacy of pregabalin as an add-on anticonvulsant medication for dogs who were refractory to conventional medications (phenobarbital and/or potassium bromide) found a decrease both in the number of seizures, and a decreased number of seizures per cluster in those dogs experiencing cluster seizures (15).

Currently, no pharmacokinetic data exist for pregabalin administered to cats. Prior to recommending pregabalin as an anticonvulsant in cats with seizure disorders, doses and dosing frequency need to be determined. Therefore, we evaluated the pharmacokinetic profile of single-dose oral pregabalin administration in healthy cats. Based on pharmacokinetic data available for humans and dogs, the hypothesis was that at 4 mg/kg dosing, plasma pregabalin concentrations would reach concentrations in cats considered therapeutic for seizure control in humans and dogs.

## Materials and methods

This study was approved by Cornell University's Institutional Animal Care and Use Committee (IACUC).

### Animals

Six healthy adult purpose-bred cats with a mean age 2.7 years (range: 9 months−4 years 8 months) weighing an average of 5.6 kg (range: 4.1–7.0 kg) were used for this study. One cat was female spayed and the remaining five were male castrated. All cats were healthy as determined by normal physical and neurologic examinations as well as unremarkable complete blood count, serum chemistry and urinalysis evaluations. Cats were group-housed (two groups of 3) in a climate-controlled indoor facility (average temperature 70–74°F, average humidity 38–53%) with a set 12 h light-dark cycle (6 am−6pm). Cats were placed in individual cages for the duration of their 36 h sampling period and then returned to the group. Food was withheld for 12 h prior to the study for sedation for catheter placement, and until they were recovered from sedation. With this exception, dry food and water were provided *ad libitum*. All cats were seen eating, drinking, urinating and defecating normally prior to, during and after the study.

### Pregabalin dosing, sample collection and processing

On the morning of the study, each cat was sedated (dexmedetomidine 15 μg/kg IM) and indwelling sampling catheters^1^ were placed in either a jugular or medial saphenous vein. Dexmedetomidine was reversed with intramuscular atipamezole (equal in volume to dexmedetomidine administered) to allow for complete recovery from sedation prior to pregabalin administration. Each cat received one oral dose of pregabalin^2^ in capsule form, individually compounded to 4 mg/kg body weight, at least 2 h after atipamezole administration. Capsules were prepared by crushing human-formulated tablets and individually weighing out the contents before re-packaging in gelatin capsules. Blood samples (2 ml/sample, total blood volume taken was 26 ml/cat) were collected at time = 0 (prior to pregabalin administration), and at 15, 30, 60, 90, 120, 180, 240, 360, 480, 720, 1,440, and 2,160 min. After sampling, catheters were flushed with heparinized saline (2 units heparin/ml of 0.9% saline). Two cats (cats #2 and #3) received subcutaneous replacement fluids following the study due to blood volume taken approximating 10% total blood volume. Cats were evaluated for any apparent drug side effects by one or more of the investigators at times of blood draws.

Samples were maintained on ice until centrifuged at 500 g for 15 min at 4°C (Allegra X-15R; Beckman Coulter Inc., Fullerton, CA, USA). Plasma was collected from these samples and frozen at −70°C until shipped on dry ice for measurement of pregabalin concentrations via high performance liquid chromatography (HPLC).

Pregabalin concentrations were measured via quantitative assay performed by a commercial laboratory^3^ using an HPLC procedure modified from a previously described method. This assay has been shown to have a lower limit of detection of 0.10 μg/mL, with a between-run precision of 12.9, 2.6, and 1.9% at concentrations of 0.1, 2.0 and 8.0 μg/mL (16).

### Pharmacokinetic analysis

Pharmacokinetic analysis was performed as previously described for dogs (14). Plasma (or serum) drug X concentration versus time data was subjected to non-compartmental analysis using computer software (Phoenix WinNonLin V7, Pharsight, Cetara). Area under the curve to infinity was determined using the log-linear trapezoidal method. The actual maximum concentration (C_Max_) occurring at time to maximum concentration (T_Max_) were recorded. Concentrations at 12 h (C12) and at the last time point collected (C_Min_) were also recorded. The slope of the terminal component of the drug-elimination time curve was based on non-linear regression. Because pregabalin was not given intravenously, the terminal component could not be confirmed to be elimination and thus both the elimination rate constant and half-life were reported as disappearance; half-life was reported as harmonic mean + pseudostandard deviation. Further, neither clearance (CL) nor volume of distribution (Vd) could be determined and are reported uncorrected for bioavailability (Vd/F or CL/F). Other parameters included addition mean residence time (MRT) and the percent of the AUC that was extrapolated from the terminal component of the curve.

Predicted steady state concentrations (μg/ml) for both 12 and 24 h dosing, including maximum steady state concentration (Css_Max_), minimum steady state concentration (Css_Min_), and average steady state concentration (Css_Av_) were extrapolated using commercially available software^4^ (http://www.SummitPK.com).

## Results

**Figure 1:**
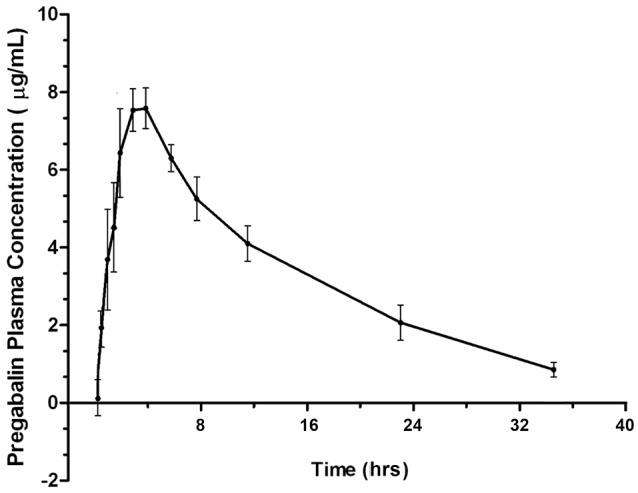
Plasma concentration vs. time curve for pregabalin (μg/ml) after 4 mg/kg oral dosing in cats.

**Figure 2:**
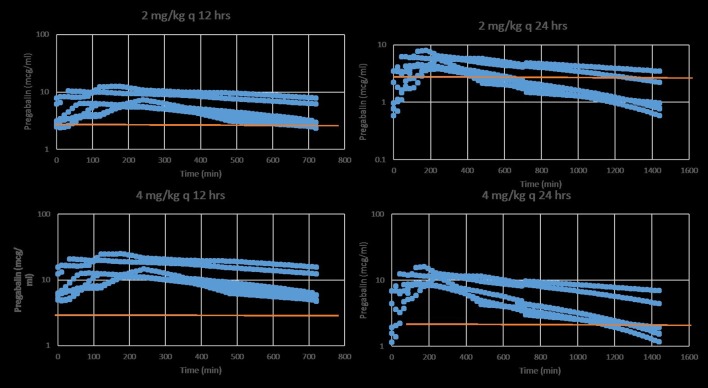
Predicted stead-state plasma pregabalin concentrations in normal healthy cats after oral administration of either 2 (**top**) or 4 mg/kg (**bottom**) q 12 (**left**) or 24 (**right**) h. The red line indicates the minimum target therapeutic concentration (2.8 mcg/ml). Based on this data, a dose of 2 mg/kg q 12 h or 4 mg/kg q 24 h is a recommended starting dose.

Four of six cats experienced moderate, transient sedation within several hours of oral pregabalin dosing, which resolved in all cats within roughly 10 h after receiving the drug, corresponding to the time when serum drug concentrations had fallen to approximately 4–5 μg/ml. The four cats with moderate sedation appeared generally unsteady on their feet and mildly obtunded, but still very interactive with the investigators. During the course of the study, all cats subjectively appeared polyphagic. One cat (#3) vomited immediately after receiving the dose of pregabalin (including the capsule). This cat was temporarily removed from the study and was re-entered 4 days later, at which time no vomiting was observed. Four of six cats had plasma pregabalin concentrations within the targeted therapeutic range for seizures in humans and dogs (2.8–8.2 μg/ml) within 90 min of drug administration. The remaining two (cats #4 and #6) had serum concentrations within this range in 180 and 120 min, respectively. All cats remained within this range until 720 min (12 h) post dosing. Figure 1 illustrates the plasma concentration vs. time curve (Mean ± SD) (14). Pharmacokinetic parameters (Mean ± SD, range) are listed in Table 1. Predicted steady-state concentrations for 12 and 24 h dosing intervals are provided in Table 2 and predicted concentrations at steady-state when pregabalin is dosed at either 2 or 4 mg/kg q 12 or 24 h is demonstrated in Figure 2. Based on this data, concentrations will remain above the minimum therapeutic concentration of 2.8 mcg/ml in most cats throughout the chosen dosing interval when dosed orally at either 2 mg/kg every 12 h or 4 mg/kg every 24 h.

**Table 1:** Pharmacokinetic parameters of pregabalin in cats after single (4 mg/kg) oral dosing.

**Parameter**	**Mean ± SD**	**Median (Range)**
Body Weight (kg)	5.6 ± 1.2	5.7 (4.1–7.0)
t_½dis_ (h)	10.4 ± 2.6	9.2 (8.3–15.1)
t_>2.8_ (h)	17.6 ± 6.2	15.5 (11.0–27.5)
C_Max_ (μg/ml)	8.3 ± 1.5	8.25 (6.4–10.0)
T_Max_ (h)	2.8 ± 1.2	3.0 (1.0–4.0)
AUC 0 → ∞ (μg-h/ml)	132 ± 44	112 (96–196)

*t_½dis_ = disappearance half-life. t_>2.8_ = time over presumed minimum therapeutic concentration of 2.8 μg/mL. C_Max_ = maximum plasma concentration. T_Max_ = time to C_Max_. AUC = area under the curve*.

**Table 2:** Predicted Steady State Plasma concentrations (μg/ml) with 4 mg/kg oral pregabalin administration in cats^*^.

**Parameter**	**12 h Interval Mean ± SD (Range)**	**24 h Interval Mean ± SD (Range)**
Css_Max_	16.2 ± 5.8 (10.4–23.0)	10.8 ± 3.6 (7.0–16.1)
Css_Min_	4.9 ± 4.1 (0.3–10.9)	2.5 ± 1.3 (1.4–4.5)
Css_Av_	9.5 ± 3.8 (5.9–15.5)	4.8 ± 1.9 (2.9–7.7)

* *Data are based on single dose administration. Values are predicted steady state plasma pregabalin concentrations with 12 or 24 h dosing intervals*.

*Css_Max_, predicted maximum plasma concentration at steady state; Css_Min_, predicted minimum plasma concentration at steady state; Css_Av_, predicted average plasma concentration at steady state*.

## Discussion

Our study provides the basic pharmacokinetic data for oral pregabalin administration in cats, and predicts a suitable dosing interval for cats. It is important to note that this data pertains to a specific compounded formulation of pregabalin, versus the commercially available tablet. These data provide the basis for prospective clinical studies examining the efficacy of pregabalin as either a sole, or add-on anticonvulsant medication for cats with seizure disorders. Mild sedation and apparent polyphagia were the only short-term side effects that we observed with pregabalin administration.

Although gabapentin has been demonstrated to be moderately effective as an add-on anticonvulsant medication in dogs (17), the pharmacokinetics of oral gabapentin in cats predict that it would require 3 mg/kg q6h dosing to reach and sustain targeted anticonvulsant plasma concentrations in humans (13). On the other hand, our study suggests that pregabalin at 4 mg/kg q24h would achieve and sustain targeted anticonvulsant concentrations. Clients are much more likely to comply with such a dosing regimen.

In our study, cats had a subjectively longer T_Max_ (2.9 h) than dogs (1.9 h) or humans (1.3 h) (we did not formally compare these). Similarly, cats had a subjectively longer t_½elim_ (10.4 h) than dogs (6.8 h) or humans (6 h) (18–21). Cats had a subjectively higher average C_Max_ (8.3 μg/ml in cats vs. 6.8 μg/ml in dogs) and a more prolonged t_½dis_ than dogs, suggesting a higher degree of drug absorption as well as a slower elimination (14, 21). Cats in our study were fasted prior to drug administration for safety associated with sedation. In humans, administering pregabalin with food reduces the peak plasma levels by 25–30% and prolongs T_Max_ to roughly 3 h, though overall AUC and plasma half-life are unaffected (5). Without a trial specifically evaluating pharmacokinetics with and without food administration in cats, we are unable to speculate about the effects of feeding. As most cats in a clinical setting would not be fasted prior to receiving the drug, it is possible that our evaluation of certain parameters may be different than if cats had been able to eat *ad libitum* during the study.

It is unclear from our study why cats might have a higher C_Max_ and t_½dis_ than dogs. Absorption of the predecessor of pregabalin (gabapentin) is thought to be due to its action as a substrate of the system L transporter, which is responsible for moving large amino acids across the intestine and is ubiquitous amongst many species. Species differences might exist in this transporter, allowing for more efficient uptake of pregabalin after ingestion in cats (i.e., decreased saturability of the transporter as compared with dogs). Although pregabalin undergoes negligible metabolism before renal excretion in humans, gabapentin in dogs undergoes significant (32-40%) metabolism to N-methylgabapentin, with no induction of hepatic microsomal enzymes (22, 23). A concurrent intravenous study of pregabalin would be necessary to determine the true bioavailability of the drug in cats, as without this it is difficult to speculate which parameters affecting half-life (clearance, volume of distribution) are responsible for the half-life differences seen between dogs and cats. However, we did not have an intravenous formulation of pregabalin. We administered capsules orally and inspected the oral cavity to ensure that the cat had swallowed the capsule. The capsules were not followed by a water flush to provide a more uniform transit time into the stomach. Had this been performed, it might have allowed for less variability in T_Max_ and C_Max_ between cats; however, administration of a water flush after every dose may be unrealistic in a long term, at-home setting where medication is administered by owners.

We administered a compounded form of the commercially available pregabalin^5^to the cats in our study, because the dosages of commercially available pregabalin would have exceeded 5 mg/kg in several of the cats, and because personal anecdotal experience suggests that most neurologists in the United States and Canada currently administer compounded pregabalin to cats to reduce the dose delivered, thereby avoiding the side effects of sedation that we observed. We cannot determine whether compounding pregabalin into capsule form affected bioavailability. However, our data more closely reflect the “real-world” situation of medicating pet cats with pregabalin. Another limitation of our investigation pertains to the fact that 5 cats were male and one was female. These were the cats available for the investigation. Ideally, a 50/50 split of male/female would potentially have allowed for evaluation of sex differences in pharmacokinetics and adverse effects.

We sedated the cats in our study with dexmedetomidine to place intravenous catheters for sampling, and then reversed the dexmedetomidine with a specific antagonist (atipamezole). Although it is possible that residual dexmedetomidine could have affected the pharmacokinetics of pregabalin, the elimination half-life of unreversed dexmedetomidine is short (approximately 60 min), so any residual dexmedetomidine would be unlikely to affect the pharmacokinetics of pregabalin. We could not maintain indwelling catheters in the cats in our study for prolonged periods prior to administering pregabalin because of logistical issues associated with housing and monitoring of instrumented cats. However, such an approach would allow investigators to examine any potential interaction or effect of reversed sedation with alpha-2 agonists on pregabalin pharmacokinetics.

People with seizure disorders have a targeted therapeutic range of pregabalin of 2.8–8.2 μg/ml (7, 20, 23). Although no therapeutic range has been established for animals, in a clinical report of pregabalin in dogs, 9/11 dogs (82%) achieved drug concentrations within this targeted range, and the two dogs that did not achieve the targeted concentrations were in the “responder” group, indicating that a lower minimum concentration might be sufficient for seizure control in some dogs (15). Cats in our study showed a mean time above this presumed therapeutic range (t_>2.8_) of 17.6 h, considerably higher than that reported in dogs (10.8 h). Consequently, we suspect that twice-daily dosing in cats will provide for plasma drug concentrations within the presumed therapeutic range.

Time from first drug administration to plasma steady-state is generally achieved in five elimination half-lives (t_½elim_); therefore, the cats in our study would have achieved a steady-state concentration in approximately 2 days. Repeated dosing (as compared to a single dose, as in our study) would result in an increasing C_Max_ and C_Min_ incrementally with each dose until steady state plasma concentrations are reached. Steady state plasma concentrations were predicted for our data using software simulation. This shows that at 4 mg/kg q12hr dosing, Css_Max_ is quite high (16.2 μg/ml) relative to the presumed therapeutic range, with a Css_Min_ still within this range (4.9 μg/ml). However, when dosing interval is increased to q24hr, Css_Max_ and Css_Av_ remain within the presumed therapeutic range (10.8 and 4.8 μg/ml, respectively), but Css_Min_ falls to 2.5 μg/ml, slightly below the presumed therapeutic range. At 4 mg/kg, once daily dosing might be adequate. However, lower doses might require q12hr dosing. This might reduce some of the side effects of the drug while maintaining targeted serum concentrations.

Our study indicates a rapid absorption and a favorable pharmacokinetic profile for pregabalin in cats. Given the moderate side effects (sedation, polyphagia) exhibited by the cats as well as the high plasma levels likely to be achieved at steady-state with the 4 mg/kg dose used in this study, a lower dose (i.e.: 1–2 mg/kg q12hr) might be a more acceptable starting dose. Further studies are required to confirm our predictions.

## Author contributions

ME: assistance in project inception, data collection, manuscript preparation; CD: project inception, data collection, manuscript preparation and editing; WS: pharmacokinetic analysis, manuscript review and editing; MR: statistical analysis, manuscript preparation and editing; DB: pharmacokinetic analysis, manuscript review and editing.

### Conflict of interest statement

The authors declare that the research was conducted in the absence of any commercial or financial relationships that could be construed as a potential conflict of interest.

## Footnotes

^1^BD Intracath, Beckton Dickinson Infusion Therapy Systems, Inc., Sandy, UT, USA.

^2^Lyrica®, Pfizer Inc., New York, NY, USA.

^3^National Medical Services Labs, Willow Grove, PA, USA.

^4^PK Solutions 2.0 Summit Research Services, Montrose, CO, USA.

^5^Lyrica®, Pfizer Inc., New York, NY, USA.

## Abbreviations

AUC: Area under the curve
C_Max_: Maximum concentration
Css_Av_: Average concentration at study state
Css_Max_: Maximum concentration at study state
Css_Min_: Minimum concentration at study state
GABA: Gamma-aminobutyric acid
HPLC: High performance liquid chromatography
t_½elim_: Elimination half-life
t_Max_: Time to maximum concentration
t_>2.8_: Time above presumed therapeutic range.
